# Identification of QTLs for Arsenic Accumulation in Maize (*Zea mays* L.) Using a RIL Population

**DOI:** 10.1371/journal.pone.0025646

**Published:** 2011-10-18

**Authors:** Dong Ding, Weihua Li, Guiliang Song, Hongyuan Qi, Jingbao Liu, Jihua Tang

**Affiliations:** 1 College of Agronomy, Key Laboratory of Physiological Ecology and Genetic Improvement of Food Crops in Henan Province, Henan Agricultural University, Zhengzhou, China; 2 College of Plant Science and Technology, Huazhong Agricultural University, Wuhan, China; Centre for Genomic Regulation, Spain

## Abstract

The Arsenic (As) concentration in different tissues of maize was analyzed using a set of RIL populations derived from an elite hybrid, Nongda108. The results showed that the trend of As concentration in the four measured tissues was leaves>stems>bracts>kernels. Eleven QTLs for As concentration were detected in the four tissues. Three QTLs for As concentration in leaves were mapped on chromosomes 1, 5, and 8, respectively. For As concentration in the bracts, two QTLs were identified, with 9.61% and 10.03% phenotypic variance. For As concentration in the stems, three QTLs were detected with 8.24%, 14.86%, and 15.23% phenotypic variance. Three QTLs were identified for kernels on chromosomes 3, 5, and 7, respectively, with 10.73%, 8.52%, and 9.10% phenotypic variance. Only one common chromosomal region between SSR marker bnlg1811 and umc1243 was detected for QTLs *qLAV1* and *qSAC1*. The results implied that the As accumulation in different tissues in maize was controlled by different molecular mechanism. The study demonstrated that maize could be a useful plant for phytoremediation of As-contaminated paddy soil, and the QTLs will be useful for selecting inbred lines and hybrids with low As concentration in their kernels.

## Introduction

Soil contamination with toxic heavy metals and metalloids, such as Arsenic (As), has become a worldwide problem. Arsenic is ubiquitously encountered in the environment because of its release in substantial amounts as a consequence of geological and/or anthropogenic activities. These activities include mining, burning of fossil fuels, use of fertilizers and agrochemicals, disposal of municipal and industrial wastes, and irrigation with contaminated water [1]–[4], especially in Asia [5]–[9]. Irrigation of vegetables and crop plants with arsenic-contaminated water, and accumulation of As by plants, causes arsenic exposure to humans through their daily diet [10]. Arsenic exposure increases the risk of certain types of human cancer, such as skin, bladder, lung, kidney, and liver cancers [11].

At a higher concentration, arsenic is also toxic to most plants. It interferes with metabolic processes and inhibits plant growth and development by arsenic induced phytotoxicity [12]. When plants are exposed to excess arsenic, either in soil or in solution culture, they exhibit toxicity symptoms, such as inhibition of seed germination [13]–[14]; decreased plant height and tillering [15]–[16]; reduction in shoot and root growth [17]–[18]; lower fruit and grain yield [12], [19]; wilting and necrosis of leaf blades [21], reduction chlorophyll content and leaf area, as well as photosynthesis [22]–[24]; and sometimes, plant death [25]–[26].

With uniform soil As concentration, there is a large variation in total As concentration in grains of different genotypes of rice [27]–[28]. Several QTLs for As concentration in rice have been identified [29]–[30], and a remarkable three-gene model of tolerance was advanced using the same population, which appears to involve epistatic interaction between three major genes [31]. Ma et al. reported that two different types of transporters (*Lsi1* and *Lsi2*) mediate transport of arsenite in rice [32], and that NIP1;2 and NIP5;1, closely related homologs of NIP1;1, were also permeable to As(III) [33]–[34]. In Arabidopsis thaliana, Pho1;1 and Pho1;4 are responsible for As(V) and phosphate uptake [35]. Pht1;1–3 harbors a semidominant allele coding for the high affinity Pi transporter PHT1;1 [36]. Recently, Sung et al. reported that a mutant, ars5 in the subunit F (PAF1) of the 26S proteasome complex was shown to exhibit an increased accumulation of arsenic and thiol compounds during arsenic stress in Arabidopsis [37].

In many countries where most maize products are not directly used as human food, they are mostly used as feedstuff for livestock and poultry; however, maize may represents the first product in the biological chain leading to cereal crops and as such, its quality is important because of the potential for accumulating toxic heavy metals and metalloids. Requejo and Tena reported that the main response of plant roots to acute inorganic arsenic toxicity is the upregulation of a set of oxidative stress related proteins [11]. However, compared to Arabidopsis, rice, and wheat, there is little research on As concentration in maize and the genetic basis for As accumulation and distribution remains unclear. The objectives of this study were to (i) dissect the rules of As accumulation and distribution in different maize tissues, and (ii) identify QTLs for As concentration variations in the tissues of maize under As accumulated paddy soil treatment.

## Results

### Performance of arsenic content in the four measured traits

In terms of As concentration in the four measured tissues, of the two parents and hybrid, the parent Huang C (P_1_) had a lower As concentration (0.630 mg kg^−1^) than parent Xu178 (0.731 mg kg^−1^) in the leaves; the As concentration in the leaves of the hybrid was 0.994 mg kg^−1^. In the bracts, the As concentration in the parent Huang C (0.089 mg kg^−1^) was higher than that in parent Xu178 (0.032 mg kg^−1^); however, for stems and kernels, the As concentrations in the parent Huang C (0.040 and 0.006 mg kg^−1^) were lower than those in parent Xu178 (0.087 and 0.011 mg kg^−1^). The data demonstrated that the As distribution in the four measured tissues were different in the two genotypes. Additionally, the As concentration of in the leaves and bracts of the hybrid indicated high and low parent heterosis, and in stems and kernels it expressed a mid-parent performance.

**Figure 1:**
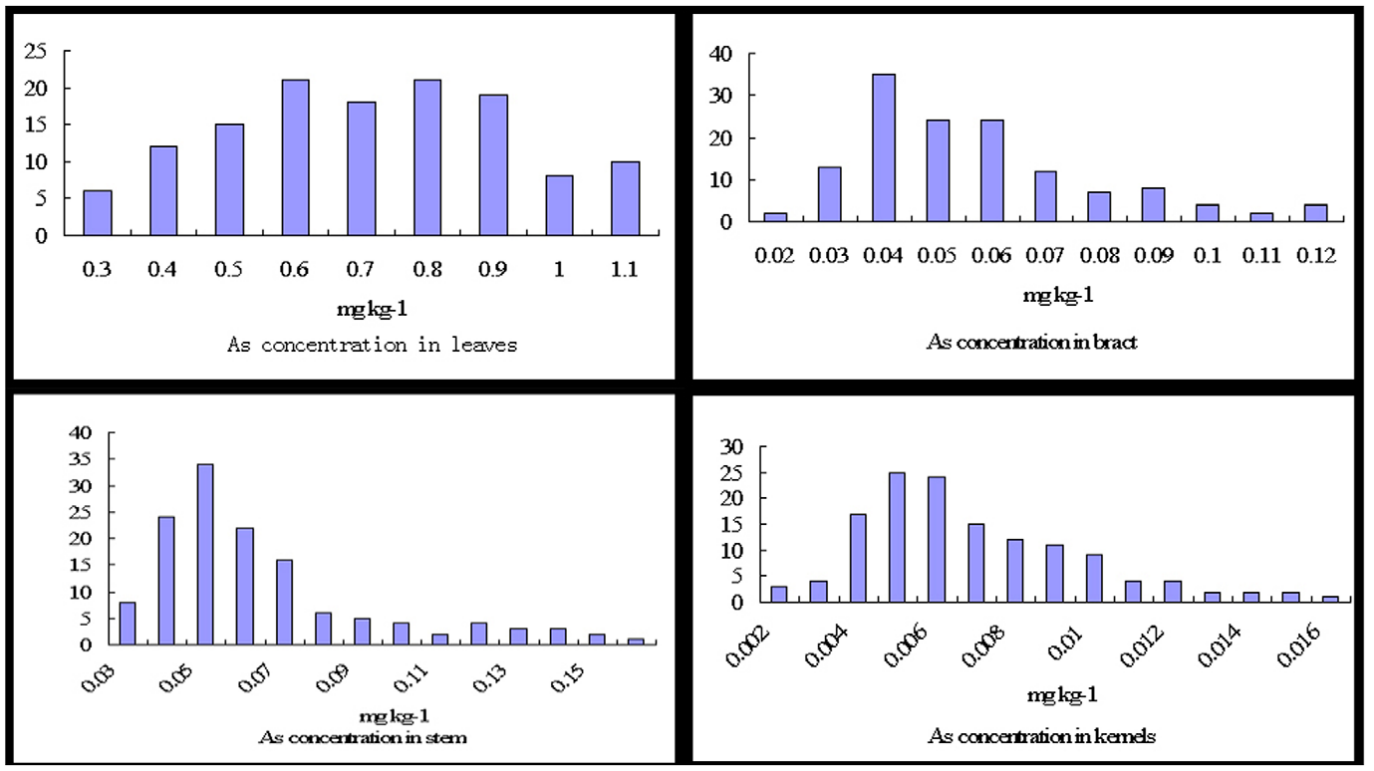
Histogram of As concentration in the four tissues of the RIL population.

The values of the As concentration in the four measured tissues in the RIL populations varied widely (Table 1, Fig. 1), and the As concentration in the four tissues of maize had significantly difference at p<0.01 level (p = 0.0004). The average As concentration of the leaves in the RIL population was 0.66±0.29 mg kg^−1^ (range 0.196 to 1.193 mg kg^−1^). For the As concentration in the stems, the average was 0.058±0.033 mg kg^−1^ (range 0.021 to 0.181 mg kg^−1^). For the As concentration in bracts, the mean was 0.051±0.029 mg kg^−1^ (range 0.015 to 0.173 mg kg^−1^), and the average As concentration in kernels was 0.0058±0.004 mg kg^−1^ (range 0.001 to 0.019 mg kg^−1^).

**Table 1:** Performance of As concentration in the four tissues of maize in the RIL population.

Population	Trait	LAC (mg kg^−1^)	BAC (mg kg^−1^)	SAC (mg kg^−1^)	KAC (mg kg^−1^)
P_1_	Mean	0.630	0.089	0.040	0.006
P_2_	Mean	0.731	0.032	0.087	0.011
F_1_	Mean	0.994	0.024	0.070	0.009
RIL	Mean	0.66±0.29	0.051±0.029	0.058±0.033	0.0058±0.004
	Range	0.196∼1.193	0.015∼0.173	0.021∼0.181	0.001∼0.019
	Skewness	0.402	1.434	1.497	2.228
	Kurtosis	−0.278	2.878	2.599	3.029

Note: LAC, As concentration in the leaves; BAC, As concentration in the bracts; SAC, As concentration in the stems; KAC, As concentration in the kernels.

For the measured trait evaluated in the RIL population under high As accumulating paddy soil, the four measured tissues had no significant relationship each other (data not shown), according to phenotypic relationship analysis. The results of variance analysis showed that the As concentration in the bracts, stems and kernels exhibited significant variations in genotypes, respectively (p<0.01 and P<0.05). The As concentration in the leaves had no significant variations in the different genotypes in the RIL population (Table 2). Amongst the different tissues of maize, the leaves had a highest As concentration, followed by stems and bracts, with kernels having the lowest As concentration.

**Table 2:** Variance analysis of the four measured tissues for As concentration in the RIL. population.

Source of variance	*F* value			
	Leaf	Bract	Stem	Kernel
Replication	0.043	0.00	0.00	0.00
Genotype	0.094	0.001^**^	0.002^**^	0.00^*^

Note:^*, **^, significant at *P*<0.05 and *P*<0.01 using F-test.

### QTL analysis for As concentration in the four tissues of maize

The genetic linkage map for the RIL population was constructed using 217 SSR markers and Mapmaker 3.0 software. It included 10 linkages, spanning a total of 2438.2 cM, with an average interval of 11.2 cM (Xie et al., 2010).

**Figure 2:**
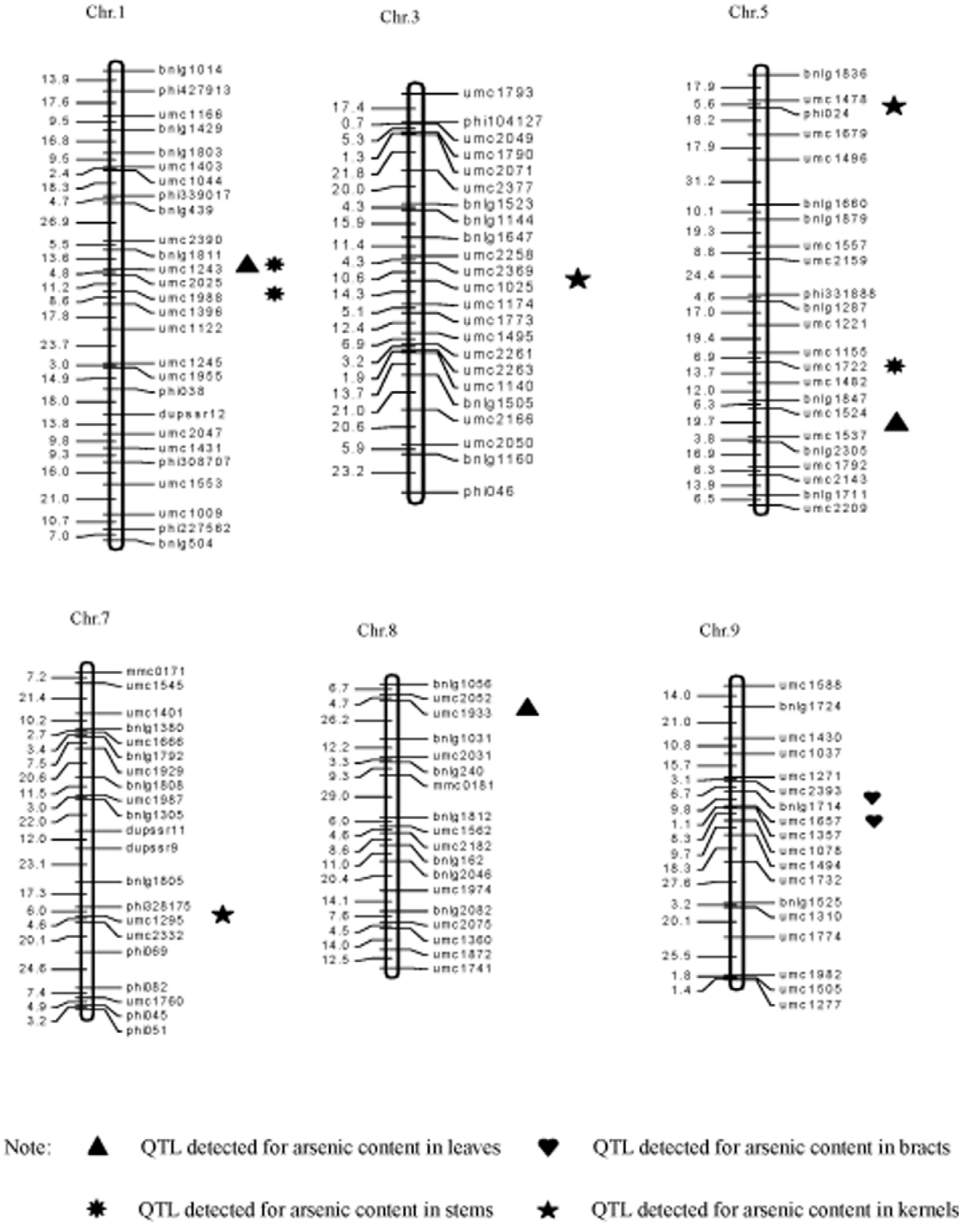
Chromosomal locations of QTLs detected for arsenic concentration for the four measured traits in maize.

Eleven different QTLs were identified for As concentration in the four measured tissues in the population under As treatment (Table 3). These QTLs were distributed on chromosomes 1, 3, 5, 7, 8, and 9 (Fig. 2). There were three different QTLs detected for As concentration in leaves. QTL *qLAC1* had a 5.62% phenotypic contribution for As concentration in the leaves, and the allele was derived from the parent Huang C. The other two QTL, *qLAC5* and *qLAC8*, explaining 5.97% and 5.62% of phenotypic variance, respectively, came from parent Xu178.

**Table 3:** QTLs detected for arsenic concentration in the four tissues in maize.

Treat	QTL^a^	Chromosome	Location	Flanking-markers	LOD^b^	A^c^	R^2^ d
Leaf	*qLAC1*	1	138.9	bnlg1811-umc1243	3.76	0.233	5.62
	*qLAC5*	5	233.5	umc1524-umc1537	3.91	−0.240	5.97
	*qLAC8*	8	11.5	umc2052-bnlg1031	3.81	−0.223	5.62
Bract	*qBAC9a*	9	81.1	bnlg1714-umc1357	3.57	0.008	9.61
	*qBAC9b*	9	98.8	umc1078-umc1494	3.25	0.008	10.03
Stem	*qSAC1a*	1	125.5	bnlg1811-umc1243	2.81	−0.010	8.24
	*qSAC1b*	1	155.1	umc1988-umc1396	4.15	0.014	14.86
	*qSAC5*	5	211.7	umc1722-bnlg1847	4.26	−0.013	15.23
Kernel	*qKAC3*	3	113.4	umc2369-umc1174	3.53	0.002	10.73
	*qKAC5*	5	18	umc1478-phi024	2.89	0.001	8.52
	*qKAC7*	7	162.3	phi328175-umc1295	3.15	−0.001	9.10

Notes:

a QTLs detected for As concentration in the four tissues of maize;

b LOD for each QTL;

c Additive effect; positive values indicate that Huang-C alleles increase rates;

d R^2^, contribution ratio.

For the As concentration in the bracts, two QTLs, *qBAC9a* and *aBAC9b*, were identified, which explained 9.61% and 10.03% phenotypic variation, respectively, with direct values of 0.008 and 0.008 mg kg^−1^ for As concentration in the bract respectively. The two increased effect QTLs both derived from the high As concentration parent, Huang C.

Three QTLs were identified for As concentration in the stems in the RIL population. Of the three QTLs, *qSAC5*, had a high contribution (15.23%) to the variance in the As concentration in the stems, with a direct 0.014 mg kg^−1^ increase in As concentration. The *qSAC5* allele was derived from the high As concentration parent Xu178. Another QTL, *qSAC1b*, which had a 14.86% phenotypic contribution for As concentration in stems, caused a direct increase of 0.013 mg kg^−1^ As concentration, was derived from the parent Huang C. The total cumulative contribution of the three QTLs to the phenotypic variance of As concentration was 38.33%.

Three QTLs were identified for As concentration in kernels in the RIL population and were located on chromosomes 3, 5 and 7, respectively. The QTLs, *qKAC3*, *qKAC5 and qKAC7*, had 10.73%, 8.52% and 9.10% contribution rates to the phenotypic variance, with direct increases of 0.002 mg kg^−1^, 0.001 mg kg^−1^, 0.001 mg kg^−1^ in As concentration in the kernels, respectively. The alleles from the parent Huang C (QTLs *qKAC3* and *qKAC5*) were associated with increased As concentration in kernels. The other QTL, *qKAC7*, was derived from the parent Xu178 with increasing effect. The total QTLs effects detected for As concentration in kernels could explain 28.35% of the phenotypic variance.

Out of the 11 QTLs detected for the As concentration trait in the maize tissues, only one common chromosomal region, between SSR markers bnlg1811 and umc1243, was found for two QTLs, *qLAC1* and *qSAC1*. The QTL detection results verified that the As concentration in the four tissues of maize had no significant relationship, and that the As concentration in the different maize tissues was possibly controlled by different genetic mechanisms.

## Discussion

### As distribution in the different tissues in plant

Food is one of the most important sources from which humans take up arsenic, and soil arsenic is the major source of As uptake by crops. The As concentrated in the grain of a cereal crop can enter the human body directly; therefore, the distribution of As concentration in different plant tissues has received close attention in previous studies. In rice, Abedin et al. observed that a very large amount of arsenic was retained in rice roots compared to its content in straw and rice grain [20], which agreed with previous studies [21], [41]–[42]. Rahman et al. reported that the order of arsenic concentrations was rice hull>branpolish>brown rice>raw rice>polished rice in two widely cultivated rice varieties [41]. Smith et al. reported that arsenic concentrations in rice tissue increased in the order grain, leaf, stem, and root [43]. In maize, Baig et al. reported that the translocation of total As in different tissues of maize were in the order of root>shoot>grain [44]. In this study, we found that the As concentration in the seed/kernels of cereal crop was lower than in the other tissues, and the trend of As concentration in different tissues was leaves>stems (shoot)bracts>kernels. The results implied that the mechanism of As accumulation and distribution in different tissues of maize is possibly related to a self detoxification mechanism of the plant.

### The genetic basis of As accumulation and distribution in the different tissues of maize

In the environment, arsenic (As) is present in both organic and inorganic forms; the inorganic species, arsenate [As (V)] and arsenite [As(III)], are more abundant in soils compared with the organic As [45]. As (V) has been shown to be taken up by the high affinity phosphate uptake system [46]–[48]. Abercrombie et al. reported that Antioxidant-related genes play prominent roles in response to arsenate. Microarray data suggest that As (V) induces genes involved in response to oxidative stress and represses transcription of genes induced by phosphate starvation [49]. As(III) uptake, on the other hand, is thought to be accomplished through aquaporins in the roots [50]. When As (V) enters the plant, a proportion of it is reduced to As (III), a process thought to lead to oxidative stress [51]. Ma et al. reported that a mutation in *Lsi2* had a much greater impact on arsenic accumulation in shoots and grain of field-grown rice than that in *Lsi1*, which suggested that the root-to-shoot translocation is the key step in controlling As accumulation in shoots [32].

In maize, Mylona et al. have shown that maize enzymes involved in reactive oxygen scavenging have increased activity and increased gene expression upon As exposure [52]. Requejo and Tena, studying protein profiles, showed that 10% of the detectable proteins in maize roots were regulated (either up- or downregulated) by As, and seven out of the 11 proteins whose identity was revealed were involved in cellular homeostasis for redox perturbation [11]. In this study, 11 QTLs for As concentration in different tissues of maize have been identified, and only two QTLs share the same chromosomal region, implying that As accumulation and distribution in different tissues is perhaps controlled by different genetic mechanisms.

### Phytoremediation of As contaminated paddy soil

Recently, arsenic contamination in the environment has aroused considerable attention [53]–[54]. The maximum acceptable concentration of arsenic in agricultural soil is 20 mg kg^−1^ [55]; however, in many areas or countries of the world, such as in Bangladesh and China, the As concentration in the paddy soil is more than the maximum acceptable concentration [56]–[58]. To decrease the As concentration in paddy soil, many types of physical and chemical remediation methods have been used. Compared to physical and chemical remediation methods, phytoremediation is an emerging, cost-effective, and noninvasive alternative or complementary technology that uses green plants to clean up heavy metals from the environment [59] . The plants used in phytoremediation display a wide range of mechanisms at the cellular level that are potentially involved in detoxification, and thus tolerance, of heavy metals and metalloids stress [60]–[61]. Crop plant species such as maize, which are cultivated with high biomass production according to well established agronomic methods, can be more interesting in phytoextraction protocols than metal hyperaccumulating plants, which are wild species with very rates of growth and biomass production [11]. To use maize in arsenic phytoextraction protocols, the mechanisms of As accumulation in the various tissues of maize might constitute appropriate selection and/or manipulation targets for improving the potential of maize in arsenic phytoremediation. In this study, we found that the leaves and stems are the major tissues of As accumulation in the RIL population grown in As contaminated soil; however, the As concentrations are lower than those in different tissues of rice, where the leaves, stems and bracts was the main biomass product in maize. Maize is the most planted crop worldwide, and has a broadly adaptability; therefore, it has good prospects in phytoremediation for renovating As contaminated soil, as proposed by Requejo & Tena [11].

### The utilization of QTLs for As concentration in maize breeding

With uniform soil As concentration, there is a large variation in total As in grains of different genotypes [27]. Both environmental and genotype differences affect As uptake and speciation in rice [28]. Wu et al. reported that Arsenic accumulation is significantly different between genotypes of rice. They also pointed out that the variation of genotypes for As accumulation and speciation would be useful for selecting genotypes to grow in areas contaminated by As [62]. Recently, Zhang et al. reported that molecular markers tightly linked to QTLs detected for As concentration could be used in the development of rice cultivars with low straw and grain As, using marker-assisted selection (MAS) [30]. Obviously, the As concentration and distribution in different tissues of maize is a typical quantitative trait; thus, the QTLs detected for As concentration in different tissues can also be used in MAS for selecting kernels with a low As concentration.

## Materials and Methods

### The arsenic content in the soil

The study was conducted to investigate the accumulation and distribution of arsenic in different tissues of maize in a RIL population, which was planted in As affected paddy soil located in Ningling county of Henan Province in China (E115°31′, N34°44′). The agricultural soil of the study area has become highly contaminated with arsenic because of the use of arsenic-rich surface water (11.02±0.95 mg kg^−1^ As, PH = 6.5) for irrigation.

### The experimental population

A population of 203 recombinant inbred lines (RIL) was constructed by a single seed descent method from a cross between two elite inbred lines, Huang-C and Xu178 [38]. In 2009, the RIL population, two parents and their hybrid (Nongda108) were evaluated in experimental fields in Ningling county, which is located in the north China and has an average temperature of 14.3°C and 640.9 mm of average rainfall per year. The field experiment followed a complete randomized plot design with three replications. Each experimental material was planted in one plot, and each plot consisted of 16 plants in a single 4 m long row, with a distance of 0.27 m between two plants. Rows were planted 0.67 m apart, allowing a density of 65250 plants per hectare. To ensure the growth of 16 plants per plot, seeds were sown in three seed-hills, and only one plant was preserved, to reduce competition among seedlings. Before planting the experimental material, the field was irrigated to ensure the seed could germinate normally.

### Analysis of As concentrations

Five consecutive plants per row, including ears, were harvested at seed physiological maturity. Oven-dried plant tissues (leaves, bracts, stems and kernels) were digested in nitric acid on a heating block (Digestion Systems of AIM500, AI Scientific, Brisbane, Australia). The concentrations of As in leaves, bracts, stems and kernels were measured three times by an atomic fluorescence spectrometry (AF-610 A, Beijing Ruili Analytical Instrument Co., Beijing, China) [30], and the average of measurements was used for further analysis. Data analyses were performed using SAS 8.0 statistical software with the PROC MIXED procedure.

### Data analysis and QTL mapping

The polymorphisms between two parents, Huang-C and Xu178, were screened using 892 pairs of simple sequence repeats (SSR) markers selected from the maize genome database (www.maizegdb.org). We chose 217 SSR markers that showed distinct polymorphisms in both parents to amplify the RIL population DNA. Molecular linkage maps were constructed using Mapmakers 3.0 at a LOD threshold less than 3.0 [39].

The composite interval mapping method and Model 6 of the Zmapqtl module of QTL Cartographer 2.0 were used to identify QTLs for the As concentration in the four tissues of maize [40]. The LOD threshold was calculated using 1000 permutations at a significance level of *P* = 0.05, with scanning intervals of 2 cM between markers and a putative QTL, and a 10 cM window. The number of marker cofactors for background control was set by forward-backward stepwise regression with five controlling markers.

## References

[1] Cullen WR, Reimer KJ (1989). Arsenic speciation in the environment.. Chemical Reviews.

[2] Roychowdhury T, Tokunaga H, Ando M (2003). Survey of arsenic and other heavy metals in food composites and drinking water and estimation of dietary intake by the villagers from an arsenic-affected area of West Bengal, India.. Science of the Total Environment.

[3] Meharg AA (2004). Arsenic in rice - understanding a new disaster for South- East Asia.. Trends in Plant Science.

[4] Rahman MA, Hasegawa H, Rahman MM, Rahman MA, Miah MAM (2007). Accumulation of arsenic in tissues of rice plant (*Oryza sativa* L.) and its distribution in fractions of rice grain.. Chemosphere.

[5] Nickson R, McArthur J, Burgess W, Ahmed KM, Ravenscroft P (1998). Arsenic poisoning of Bangladesh groundwater.. Nature.

[6] Chowdhury UK, Biswas BK, Chowdhury TR, Samanta G, Mandal BK (2000). Groundwater arsenic contamination in Bangladesh and West Bengal, India.. Environmental Health Perspectives.

[7] Smedley PL, Kinniburgh DG (2002). A review of the source, behaviour and distribution of arsenic in natural waters.. Applied Geochemistry.

[8] Huang RQ, Gao SF, Wang WL, Staunton S, Wang G (2006). Soil arsenic availability and the transfer of soil arsenic to crops in suburban areas in Fujian Province, southeast China.. Science of the Total Environment.

[9] Williams PN, Islam MR, Adomako EE, Raab A, Hossain SA (2006). Increase in rice grain arsenic for regions of Bangladesh irrigating paddies with elevated arsenic in groundwaters.. Environmental Science and Technology.

[10] Alam MGM, Snow ET, Tanaka A (2003). Arsenic and heavy metal contamination of vegetables grown in Samta village, Bangladesh.. Science of the Total Environment.

[11] Requejo R, Tena M (2005). Proteome analysis of maize roots reveals that oxidative stress is a main contributing factor to plant arsenic toxicity.. Phytochemistry.

[12] Marin AR, Masscheleyn PH, Patrick WH (1993). Soil redox-pH stability of arsenic species and its influence on arsenic uptake by rice.. Plant and Soil.

[13] Abedin MJ, Meharg AA (2002). Relative toxicity of arsenite and arsenate on germination and early seedling growth of rice (*Oryza sativa* L.).. Plant and Soil.

[14] Shri M, Kumar S, Chakrabarty D, Trivedi PK, Mallick S (2009). Effect of arsenic on growth, oxidative stress, and antioxidant system in rice seedlings.. Ecotoxicology and Environmental Safety.

[15] Carbonell-Barrachina AA, Burlo-Carbonell F, Mataix-Beneyto J (1995). Arsenic uptake, distribution and accumulation in tomato plants: effect of arsenic on plant growth and yield.. Journal of plant Nutrient.

[16] Kang LJ, Li XD, Liu JH, Zhang XY (1996). The effect of arsenic on the growth of rice and residues in a loam paddy soil.. Journal of Jilin Agricultural University.

[17] Cox MS, Bell PF, Kovar JL (1996). Different tolerance of canola to arsenic when grown hydroponically or in soil.. Journal of plant Nutrient.

[18] Carbonell-Barrachina AA, Aarabi MA, Delaune RD, Gambrell RP, Patrick WH (1998). The influence of arsenic chemical form and concentration on Spartina patens and Spartina alterniflora growth and tissue arsenic concentration.. Plant Soil.

[19] Tsutsumi M (1980). Intensification of arsenic toxicity to paddy rice by hydrogen sulphide and ferrous iron I. Induction of bronzing and accumulation in rice by arsenic.. Soil Science & Plant Nutrition.

[20] Abedin MJ, Cottep-Howells J, Meharg AA (2002). Arsenic uptake and accumulation in rice (*Oryza sativa* L.) irrigated with contaminated water.. Plant and Soil.

[21] Odanaka Y, Tsuchiya N, Matano O, Goto S (1987). Absorption, translocation and metabolism of the arsenical fungicides, iron methanearsonate and ammonium iron methanearsonate, in rice plants.. Journal of Pesticide Science.

[22] Marin AR, Pezeshki SR, Masscheleyn PH, Choi HS (1993). Effect of dimethylarsinic acid (DMAA) on growth, tissue arsenic and photosynthesis of rice plants.. Journal of Plant Nutrient.

[23] Knauer K, Behra R, Hemond H (1999). Toxicity of inorganic and methylated arsenic to algal communities from lakes along an arsenic contamination gradient.. Aquatic Toxicology.

[24] Liu XL, Zhang SZ, Shan XQ, Zhu YG (2005). Toxicity of arsenate and arsenite on germination, seedling growth and amylolytic activity of wheat.. Chemosphere.

[25] Marin AR, Masscheleyn PH, Patrick WH (1992). The influence of chemical form and concentration of arsenic on rice growth and tissue arsenic concentration.. Plant and Soil.

[26] Rahman MA, Hasegawa H, Rahman MM, Islam MN, Miah MAM (2007). Effect of arsenic on photosynthesis, growth and yield of five widely cultivated rice (*Oryza sativa* L.) varieties in Bangladesh.. Chemosphere.

[27] Liu WJ, Zhu YG, Hu Y, Williams PH, Gault AG (2006). Arsenic sequestration in iron plaque, its accumulation and speciation in mature rice plants (*Oryza sativa* L.).. Environmental Science and Technology.

[28] Norton GJ, Duan G, Dasgupta T, Islam RM, Lei M (2009). Environmental and genetic control of arsenic accumulation and speciation in rice grain: comparing a range of common cultivars grown in contaminated sites across Bangladesh, China, and India.. Environmental Science and Technology.

[29] Dasgupta T, Hossain SA, Meharg AA, Price AH (2004). An arsenate tolerance gene on chromosome 6 of rice.. New Phytologist.

[30] Zhang J, Zhu YG, Zeng DL, Cheng WD, Qian Q (2008). Mapping quantitative trait loci associated with arsenic accumulation in rice (*Oryza sativa*).. New Phytologist.

[31] Norton GJ, Nigar M, Williams PN, Dasgupta T, Meharg AA (2008). Rice-arsenate interactions in hydroponics: a three-gene model for tolerance.. Journal of Experimental Botany.

[32] Ma JF, Yamaji N, Mitani N, Xu XY, Su YH (2008). Transporters of arsenite in rice and their role in arsenic accumulation in rice grain.. PNAS.

[33] Tanaka TK, Mitani N, Ma JF, Maeshima M, Fujiwara T (2008). NIP1;1, an Aquaporin Homolog, Determines the Arsenite Sensitivity of *Arabidopsis thaliana*.. The Journal of Biological Chemistry.

[34] Kamiya T, Tanaka M, Mitani N, Ma JF, Maeshima M (2009). NIP1;1, an Aquaporin Homolog, Determines the Arsenite Sensitivity of Arabidopsis thaliana.. The Journal of biological chemistry.

[35] Shin H, Shin HS, Dewbre GR, Harrison MJ (2004). Phosphate transport in Arabidopsis: Pht1;1 and Pht1;4 play a major role in phosphate acquisition from both low− and high-phosphate environments.. The Plant Journal.

[36] Catarecha P, Segura MD, Franco-Zorrilla JM, García-Ponce B, Lanza M (2007). A Mutant of the Arabidopsis Phosphate Transporter PHT1;1 Displays Enhanced Arsenic Accumulation.. The Plant Cell.

[37] Sung DY, Kim TH, Komives EA, Mendoza-Cózatl DG, Schroeder JI (2009). *ARS5* is a component of the 26S proteasome complex, and negatively regulates thiol biosynthesis and arsenic tolerance in Arabidopsis.. The Plant Journal.

[38] Xie HL, Ding D, Cui ZT, Wu X, Hu YM (2010). Genetic analysis of the related traits of flowering and silk for hybrid seed production in maize.. Gene & Genomics.

[39] Lander ES, Green P, Abrahamson J, Barlow A, Daly MJ (1987). MAPMAKER: an interactive computer package for constructing primary genetic linkage maps of experimental and natural populations.. Genomics.

[40] Zeng ZB (1994). Precision mapping of quantitative trait loci.. Genetics.

[41] Meharg AA, Abedin MJ, Rahman MM, Feldmann J, Cotter Howells J (2001). Arsenic uptake and metabolism in Bangladesh varieties.. Book of Abstracts, Arsenic in the Asia-Pacific Region-Managing Arsenic for Our Future.

[42] Rahman MA, Hasegawa H, Rahman MA, Rahman MM, Miah MAM (2006). Influence of cooking method on arsenic retention in cooked rice related to dietary exposure.. Science of the Total Environment.

[43] Smith E, Juhasz AL, Weber J, Naidu R (2008). Arsenic uptake and speciation in rice plants grown under greenhouse conditions with arsenic contaminated irrigation water.. Science of the Total Environment.

[44] Baig JA, Kazi TG, Shah AQ, Arain MB, Afridi HI (2010). Evaluating the accumulation of arsenic in maize (*Zea mays* L.) plants from its growing media by cloud point extraction.. Food and Chemical Toxicology.

[45] Takamatsu T, Aoki H, Yoshida T (1982). Determination of arsenate, arsenite, monomethylarsonate, and dimethylarsinate in soil polluted with arsenic.. Soil Science.

[46] Ullrich-Eberius CI, Sanz A, Novacky AJ (1989). Evaluation of arsenate- and vanadate-associated changes of electrical membrane potential and phosphate transport in Lemna gibba G1.. Journal of Experimental Botany.

[47] Scott N, Hatlelid KM, MacKenzie NE, Carter DE (1993). Reactions of arsenic(III) and arsenic(V) species with glutathione.. Chemical Research in Toxicology.

[48] Quaghebeur M, Rengel Z (2003). The distribution of arsenate and arsenite in shoots and roots of Holcus lanatus is influenced by arsenic tolerance and arsenate and phosphate supply.. Plant Physiology.

[49] Abercrombie JM, Halfhill MD, Ranjan P, Rao MR, Saxton AM (2008). Transcriptional responses of Arabidopsis thaliana plants to As (V) stress.. BMC Plant Biology.

[50] Meharg AA, Jardine L (2003). Arsenite transport into paddy rice (Oryza sativa) roots.. New Phytologist.

[51] Hartley-Whitaker J, Ainsworth G, Meharg AA (2001). Copper and arsenate-induced oxidative stress in Holcus lanatus L. clones with differential sensitivity.. Plant, Cell and Environment.

[52] Mylona PV, Polidoros AN, Scandalios JG (1998). Modulation of antioxidant responses by arsenic in maize.. Free Radical Biology and Medicine.

[53] Stone R (2008). Arsenic and paddy rice: a neglected cancer risk.. Nature.

[54] Zhu YG, Sun GX, Lei M, Teng M, Liu YX (2008). High percentage inorganic arsenic content of mining impacted and nonimpacted Chinese rice.. Environmental Science and Technology.

[55] Kabata-Pendias A, Pendias H (1992). Trace elements in soil and plants, 2nd ed.

[56] Smith AH, Hopenhaynrich C, Bates MN, Goeden HM, Hertzipicciotto I (1992). Cancer risks from arsenic in drinking water.. Environmental Health Perspectives.

[57] Ullah SM (1998). Arsenic contamination of ground water and irrigated soils of Bangladesh..

[58] Liao XY, Chen TB, Xie H, Liu YR (2005). Soil as contamination and its risk assessment in areas near the industrial districts of Chenzhou City, Southern China.. Environment International.

[59] Pilon-Smits E (2005). Phytoremediation.. Annual Review of Plant Biology.

[60] Clemens S, Kim EJ, Neumann D, Schroeder JI (1999). Tolerance to toxic metals by a gene family of phytochelatin synthases from plants and yeast.. The EMBO Journal.

[61] Cobbett C, Goldsbrough P (2002). Phytochelatins and metallothioneins: roles in heavy metal detoxification and homeostasis.. Annual Review of Plant Biology.

[62] Wu C, Ye ZH, Shu WS, Zhu YG, Wong MH (2011). Arsenic accumulation and speciation in rice are affected by root aeration and variation of genotypes.. Journal of Experimental Botany.

